# Supplementary material for: Modelling Skylarks (Alauda arvensis) to Predict Impacts of Changes in Land Management and Policy: Development and Testing of an Agent-Based Model
Source: PLoS One. 2013 Jun 6;8(6):e65803. doi: 10.1371/journal.pone.0065803 (PMC3675089; doi:10.1371/journal.pone.0065803)
Supplement: Supporting Information S4 — The skylark ODdox as a zipped archive. (ZIP) [file pone.0065803.s004.zip › Skylark_ODdox/class_scalable_position_map.html]

ALMaSS Skylark ODdox: ScalablePositionMap Class Reference


ALMaSS Skylark ODdox
 2.0


- Main Page
- Related Pages
- Classes
- Files

- Class List
- Class Index
- Class Hierarchy
- Class Members

Public Member Functions |
Protected Attributes

ScalablePositionMap Class Reference

Used to map locations of individuals for density estimates. Each cell can only contain one value (it is binary).
More...

`#include <positionmap.h>`

List of all members.

|  |  |
| --- | --- |
| Public Member Functions | |
| void | ClearMapValue (unsigned a\_x, unsigned a\_y) |
| int | GetMapDensity (unsigned a\_x, unsigned a\_y, unsigned a\_range) |
| bool | GetMapPositive (unsigned a\_x, unsigned a\_y, unsigned a\_range) |
| bool | GetMapValue (unsigned a\_x, unsigned a\_y) |
|  | Get the value a x,y. |
|  | ScalablePositionMap (Landscape \*L, int a\_ScaleFactor) |
| void | SetMapValue (unsigned a\_x, unsigned a\_y) |
|  | ~ScalablePositionMap () |
| Public Member Functions inherited from SimplePositionMap | |
|  | SimplePositionMap () |
|  | SimplePositionMap (unsigned a\_size) |
|  | SimplePositionMap (Landscape \*L) |
| virtual | ~SimplePositionMap () |

|  |  |
| --- | --- |
| Protected Attributes | |
| int | m\_ScaleFactor |
|  | A binary scaling factor ( 1 = 2x2m 2=4x4m 3 =8x8m etc.. ) |

|  |  |
| --- | --- |
| Additional Inherited Members | |
| Public Attributes inherited from SimplePositionMap | |
| unsigned int | m\_maxx |
| unsigned int | m\_maxy |
| bool \* | m\_TheMap |

---

## Detailed Description

Used to map locations of individuals for density estimates. Each cell can only contain one value (it is binary).

---

## Constructor & Destructor Documentation

|  |  |  |  |
| --- | --- | --- | --- |
| ScalablePositionMap::ScalablePositionMap | ( | Landscape \* | *L*, |
|  |  | int | *a\_ScaleFactor* |
|  | ) |  |  |

References SimplePositionMap::m\_maxx, SimplePositionMap::m\_maxy, m\_ScaleFactor, SimplePositionMap::m\_TheMap, Landscape::SupplySimAreaHeight(), and Landscape::SupplySimAreaWidth().

: SimplePositionMap()

{

m\_ScaleFactor = a\_ScaleFactor;

m\_maxx=(L->SupplySimAreaWidth()) >> m\_ScaleFactor;

m\_maxy=(L->SupplySimAreaHeight())>> m\_ScaleFactor;

m\_TheMap = new bool[ m\_maxx\*m\_maxy ];

for (unsigned y=0; y<m\_maxy; y++)

{

for (unsigned x=0; x<m\_maxx; x++)

{

m\_TheMap[x+m\_maxy\*y] = false;

}

}

}

|  |  |  |  |  |
| --- | --- | --- | --- | --- |
| ScalablePositionMap::~ScalablePositionMap | ( |  | ) |  |

{

;

}

---

## Member Function Documentation

|  |  |  |  |
| --- | --- | --- | --- |
| void ScalablePositionMap::ClearMapValue | ( | unsigned | *a\_x*, |
|  |  | unsigned | *a\_y* |
|  | ) |  |  |

inlinevirtual

Reimplemented from SimplePositionMap.

References SimplePositionMap::m\_maxy, m\_ScaleFactor, and SimplePositionMap::m\_TheMap.

{

a\_x = a\_x >> m\_ScaleFactor;

a\_y = a\_y >> m\_ScaleFactor;

m\_TheMap[a\_x+a\_y\*m\_maxy]=false;

}

|  |  |  |  |
| --- | --- | --- | --- |
| int ScalablePositionMap::GetMapDensity | ( | unsigned | *a\_x*, |
|  |  | unsigned | *a\_y*, |
|  |  | unsigned | *a\_range* |
|  | ) |  |  |

inlinevirtual

Reimplemented from SimplePositionMap.

References SimplePositionMap::m\_maxy, m\_ScaleFactor, and SimplePositionMap::m\_TheMap.

{

int dens=0;

a\_x = a\_x >> m\_ScaleFactor;

a\_y = a\_y >> m\_ScaleFactor;

a\_range = a\_range >> m\_ScaleFactor;

for (unsigned x = a\_x; x<a\_x+a\_range; x++)

{

for (unsigned y = a\_y; y<a\_y+a\_range; y++)

if (m\_TheMap[x+y\*m\_maxy]) dens++;

}

return dens;

}

|  |  |  |  |
| --- | --- | --- | --- |
| bool ScalablePositionMap::GetMapPositive | ( | unsigned | *a\_x*, |
|  |  | unsigned | *a\_y*, |
|  |  | unsigned | *a\_range* |
|  | ) |  |  |

inlinevirtual

Reimplemented from SimplePositionMap.

References SimplePositionMap::m\_maxy, m\_ScaleFactor, and SimplePositionMap::m\_TheMap.

{

a\_x = a\_x >> m\_ScaleFactor;

a\_y = a\_y >> m\_ScaleFactor;

a\_range = a\_range >> m\_ScaleFactor;

for (unsigned x = a\_x; x<a\_x+a\_range; x++)

{

for (unsigned y = a\_y; y<a\_y+a\_range; y++)

if (m\_TheMap[x+y\*m\_maxy]) return true;

}

return false;

}

|  |  |  |  |
| --- | --- | --- | --- |
| bool ScalablePositionMap::GetMapValue | ( | unsigned | *a\_x*, |
|  |  | unsigned | *a\_y* |
|  | ) |  |  |

inlinevirtual

Get the value a x,y.

We assume that the calling code uses maximum resolution, and that all scaling is done here. So the call is e.g. in m, but the return value is calculated per the m\_ScaleFactor.

Parameters:
:   |  |  |
    | --- | --- |
    | x | and y coordinates in the raw system metric (e.g. metres). |

Returns:
:   bool - whether the cell specified is occupied (true) or not (false)

Reimplemented from SimplePositionMap.

References SimplePositionMap::m\_maxy, m\_ScaleFactor, and SimplePositionMap::m\_TheMap.

{

a\_x = a\_x >> m\_ScaleFactor;

a\_y = a\_y >> m\_ScaleFactor;

return m\_TheMap[a\_x+a\_y\*m\_maxy];

}

|  |  |  |  |
| --- | --- | --- | --- |
| void ScalablePositionMap::SetMapValue | ( | unsigned | *a\_x*, |
|  |  | unsigned | *a\_y* |
|  | ) |  |  |

inlinevirtual

Reimplemented from SimplePositionMap.

References SimplePositionMap::m\_maxy, m\_ScaleFactor, and SimplePositionMap::m\_TheMap.

{

a\_x = a\_x >> m\_ScaleFactor;

a\_y = a\_y >> m\_ScaleFactor;

m\_TheMap[a\_x+a\_y\*m\_maxy]=true;

}

---

## Member Data Documentation

int ScalablePositionMap::m\_ScaleFactor

protected

A binary scaling factor ( 1 = 2x2m 2=4x4m 3 =8x8m etc.. )

Referenced by ClearMapValue(), GetMapDensity(), GetMapPositive(), GetMapValue(), ScalablePositionMap(), and SetMapValue().

---

The documentation for this class was generated from the following files:

- positionmap.h
- PositionMap.cpp


- ScalablePositionMap
- Generated on Thu Jan 10 2013 13:15:37 for ALMaSS Skylark ODdox by
   1.8.1.1
